# The impact of primary tumor location of colorectal cancer on the survival outcome of patients with brain metastasis: a systematic review and meta-analysis

**DOI:** 10.1186/s40001-025-03614-z

**Published:** 2025-12-10

**Authors:** Tsung-Chiao Tsai, Hany Atwan, Chaeseong Yim, Junmin Song, Roha Saeed Memon, Muhammad Fahimuddin, Changlin Gong, Yu Chang, Abhishek Kumar

**Affiliations:** 1 Department of Surgery, National Cheng Kung University Hospital, College of Medicine, National Cheng Kung University, Tainan, Taiwan; 2 Faculty of Medicine, Assiut University, Assiut, Egypt; 3 Department of Medicine, New York Medical College, Metropolitan Hospital Center, New York, NY USA; 4 Department of Medicine, Jacobi Medical Center, Albert Einstein College of Medicine, Bronx, NY USA; 5 Department of Hematology and Oncology, Jacobi Medical Center, Albert Einstein College of Medicine, Bronx, NY USA

**Keywords:** Colorectal cancer, Brain metastasis, Left colon cancer, Right colon cancer, Rectal cancer

## Abstract

**Objectives:**

Several prior studies have shown that in metastasized colorectal cancer, the primary cancer’s location influences patients’ survival rates, with left-sided colorectal cancer being associated with longer survival than right-sided colorectal cancer. This study aimed to explore the influence of the primary location of colorectal cancer on survival following brain metastasis.

**Methods:**

To address this clinical question, we conducted a systematic review and meta-analysis. We included studies focused on patients diagnosed with brain metastasis from colorectal cancer. These studies reported survival outcomes based on different primary tumor sites (right versus left and colon versus rectum). The primary outcome was to aggregate the hazard ratio (HR) of left-sided colorectal cancer when metastasized to the brain compared with right-sided colorectal cancer. The secondary outcome was to aggregate the HR of rectal cancer when it metastasized to the brain compared with colon cancer.

**Results:**

Ten studies with a total of 1792 patients were included in the meta-analysis, and combined HR was calculated. Left-sided colon cancer showed higher overall survival compared with right-sided colon cancer when metastasized to the brain (HR: 0.71, 95% CI: 0.54–0.94, *I*^2^ = 0%). Rectal cancer did not show a statistically significant difference in overall survival compared with colon cancer (HR: 0.75, 95% CI: 0.40–1.41, *I*^2^ = 81%).

**Conclusions:**

Concordant with lung and liver metastases, the primary location of colorectal cancer influenced overall survival when metastasized to the brain. Left-sided colon cancer demonstrated higher overall survival than right-sided colon cancer.

**Supplementary Information:**

The online version contains supplementary material available at 10.1186/s40001-025-03614-z.

## Introduction

Colorectal cancer (CRC) is one of the most common cancers in the U.S., regarded as the top third most common malignancy in males and the top fifth most common malignancy in females, with 25–30% of the patients diagnosed with stage IV CRC initially [1]. The landscape of therapeutic strategies for this disease has significantly evolved, leading to substantial improvements in the overall survival (OS) rates of individuals with CRC [2]. Nevertheless, despite these promising advancements, this positive trend in survival rates has been offset by a notable surge in the incidence of distant metastases, particularly highlighting the emergence of brain metastases (BM) among patients [3]. The incidence of BM in CRC ranges from 0.06% to 4% [2, 4]. This phenomenon underscores the complex nature of CRC progression and prompts a thorough exploration of the underlying dynamics. [3]

In metastatic CRC, the primary tumor site, whether it is the left or right colon, is a critical factor that significantly influences disease progression and overall patient outcomes [4]. The anatomic location of the primary tumor goes beyond being a mere starting point; it actively shapes the behavior of the disease, affects responses to treatments, and ultimately dictates the patient's prognosis [5]. For example, left-sided primary CRC has shown an OS benefit in cases of lung metastasis [6] and liver metastasis [7]. The present study was designed to address this gap by systematically reviewing and quantitatively synthesizing available evidence. Specifically, the study population comprised patients with CRC who developed BMs (population), comparing those with right-sided versus left-sided or rectal primary tumors (intervention/exposure and comparison) with OS following BM as the primary outcome (outcome). This investigation aimed to clarify whether the site of the primary CRC influences survival after the onset of BM.

## Materials and methods

We performed a systematic review and meta-analysis according to the guidelines outlined in the Cochrane Handbook for Systematic Reviews and Interventions [8]. The reporting of our results adhered to the Preferred Reporting Items for Systematic Reviews and Meta-Analyses guidelines (Supplementary Table S1). Our study was registered on the PROSPERO online platform to ensure transparency and accessibility of our research protocol (CRD42023458044).

### Study selection

We conducted a comprehensive search in the Cochrane Library, Embase, and Medline electronic databases, covering the period from inception to February 2024. No language restrictions were applied during the search process to ensure inclusivity. Two investigators (H.A. and Y.C.) independently performed the search and identified relevant studies for potential inclusion. Any discrepancies were resolved through consensus between the investigators or by consulting a senior reviewer (K.Y.C.). Additional details regarding the search strategy and specific search terms can be found in Supplementary Table S2.

### Eligibility criteria

Studies satisfying all of the following criteria were included in the analysis: (1) studies designed as prospective or retrospective cohort studies, or case–control studies, whereas case reports, editorials, letters to the editor, review articles, and conference abstracts were excluded; (2) studies specifically focusing on patients diagnosed with BM originating from CRC; (3) studies reporting comparative survival outcomes according to the primary tumor site in colorectal cancer with brain metastasis (CRCBM); and (4) studies providing or allowing calculation of hazard ratios (HRs) for survival time from BM diagnosis to death. Only studies with sufficient and compatible data were included in the quantitative synthesis. Studies lacking essential information, such as unavailable HRs, incomplete outcome reporting, or inconsistent grouping, were excluded from the meta-analysis because a reliable estimation of survival effects was not feasible. Therefore, exclusion was based on data insufficiency rather than study quality or study results.

### Data extraction

The data extraction process was conducted independently by two investigators (H.A. and Y.C.). The following information was extracted from eligible studies: the last name of the first author, publication year, inclusion period, study type, database, patient inclusion criteria, treatment detail, primary tumor site classification, median OS, HR, and 95% confidence interval (CI). Any discrepancies were resolved through consensus between the investigators or by consulting a senior reviewer (J.S.).

### Quality assessment

To assess the risk of bias in the included literature, we utilized the Risk of Bias In Non-randomized Studies of Interventions (ROBINS-I) tool [9]. Two investigators (H.A and C.G.) independently conducted the critical appraisal of the included studies by using this tool. When disagreements between the assessors occurred, a third investigator (J.S.) was consulted to reach a consensus on the item in question.

### Statistical analysis

Statistical analyses were conducted by using the meta package in R software (Supplementary S3). To evaluate the relative survival outcomes of patients with CRCBM from different primary tumor sites, we acquired HRs for OS based on the survival analysis results from the included studies. These HRs were then pooled with the inverse variance method. The meta-analysis was performed using a random-effects model with a restricted maximum likelihood method, and the effect sizes were presented alongside their corresponding 95% CIs. Heterogeneity among the included studies was assessed using the I^2^ statistics proposed by Higgins and Thompson [10]. An I^2^ value of less than 25% indicated low heterogeneity, whereas values between 25 and 50% denoted moderate heterogeneity. An I^2^ value exceeding 50% indicated high heterogeneity.

## Results

### Study selection and characteristics

**Fig. 1:**
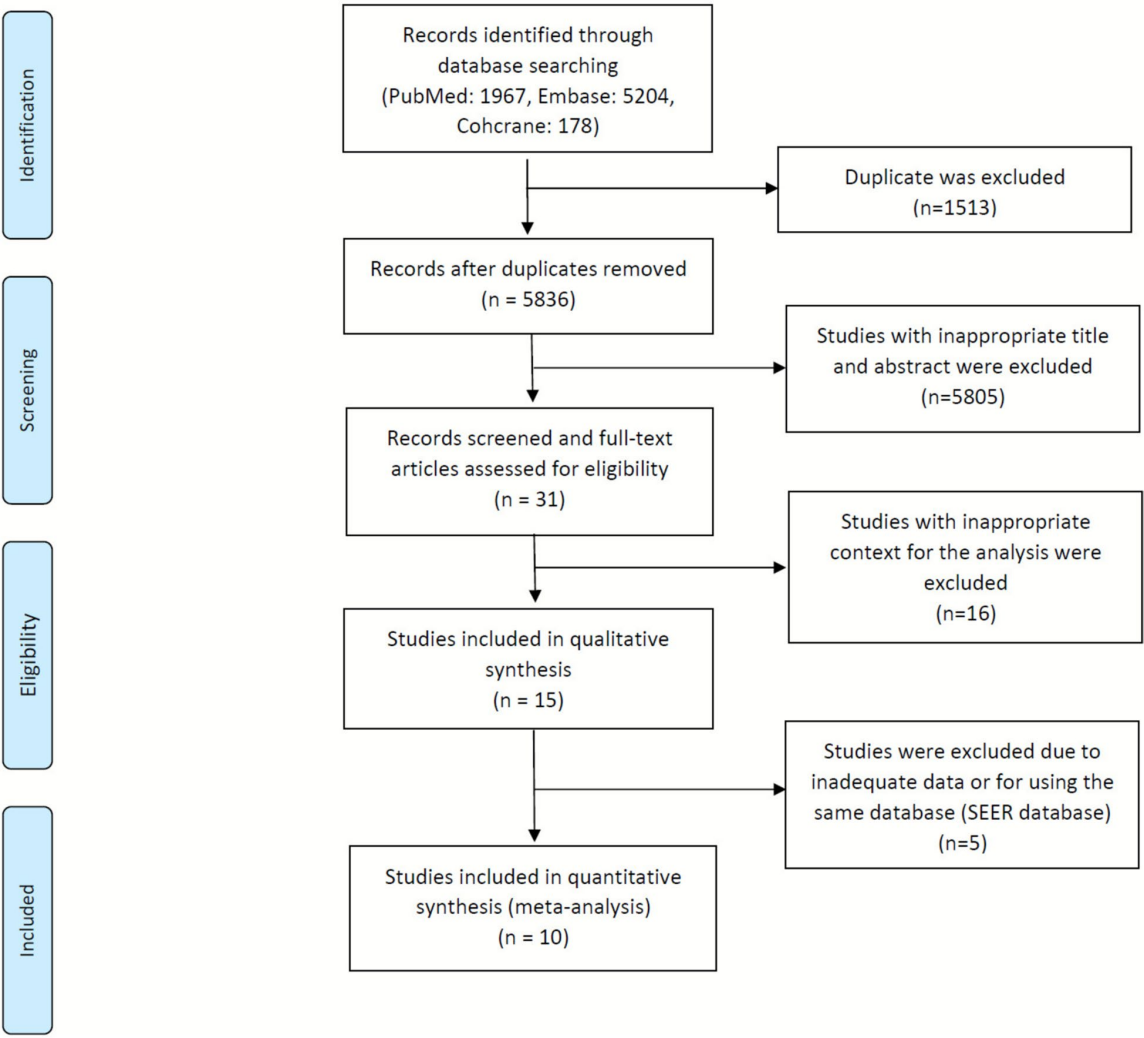
PRISMA diagram of study inclusion

Our search identified 7,349 records from the Cochrane Library, Embase, and MEDLINE databases. After removing 1,513 duplicates and excluding 5,836 irrelevant references, 31 full-text articles were assessed for eligibility. Fifteen retrospective studies published between 1996 and 2023 met the inclusion criteria and were included in the systematic review [11–25]. Among them, five studies were excluded from the meta-analysis due to insufficient or incompatible data (e.g., absence of HRs or differing survival endpoints). For studies derived from the SEER database, only Chen et al. and Yang et al. were retained to avoid data duplication, as they analyzed distinct comparisons and outcome measures. Ten studies were included in the quantitative synthesis (Fig. 1), and key study characteristics are summarized in Table 1.

**Table 1:** Characteristics of the included studies

Study	Inclusion period	Total patients (n)	Male/female (%)	Mean age (range)	Database	Patient inclusion criteria	Treatment after BM	Primary tumor site classification	Median survival time (months)
Aprile 2009	1998–2006	30	18 M / 12 F	66 (34–82)	Single Center: University Hospital of Udine	CRC patients undergoing neurosurgical treatment for BM	Surgery with or without postoperative RT	Colon: 17 (56.7%). Rectal: 9 (30%). Unknown: 4 (14.3%)	Median OS: 167 days (8–682). Postsurgical radiotherapy: 229 days. No postsurgical radiotherapy: 143 days
Bergen 2021	1988–2017	281	172 M / 109 F	61 (33–89)	Single Center: Medical University of Vienna	Patients being treated with CRC presented with BM	WBRT: 41 (14.9%) Stereotactic radiosurgery 118 (42.9%) Resection 109 (39.6%) Best supportive care: 7 (2.5%)	Left colon: 239 (85.1%). Right colon: 42 (14.9%)	Median OS: 5 (0–76) months
Bonadio 2021	2008–2019	247	124 M / 123 F	63 (22–93)	Single Center: Instituto do Câncer do Estado de São Paulo- ICESP-HC/FMUSP	Patients with histological confirmation of CRC and BM confirmation by imaging	BM surgery alone: 43 (17.4%) BM surgery with RT: 58 (23.5%) RT alone: 76 (30.8%) - Whole-brain radiation therapy: 104 (42.1%) - Stereotactic radiosurgery: 26 (10.5%) - Both: 4 (1.6%)	Left colon: 193 (78%) Right colon: 47 (19%)	Median OS: 2.9 After the BM diagnosis: - Six months 29% of patients (95% CI, 23–25) -One year: 13.5% (95% CI, 9.2–18.6)
Boysen 2020	2000–2013	235	117 M / 118 F	65 (IQR: 57–71)	Danish Cancer Registry	Patients with BM from CRC who have undergone surgical resection of BM or had received cranial SRT	Surgery Only: 158(68.4%). SBRT only: 51 (20.9%). Both: 26 (10.7%)	Colon: 120 (51.1%). Rectal: 115 (48.9%)	Median OS: patient receiving localized treatment for BM: 9.6 months (95% CI: 7.2–10.8)
Byrne 2011	1987–2009	49	29 M / 20 F	61 (30–83)	Single Center: Basingstoke & North Hampshire NHS Foundation Trust	Patients with BM from CRC and undergoing primary or repeat hepatic resection for colorectal liver metastases	Dexamethasone only: 8 Dexamethasone + shunt surgery to relieve hydrocephalus: 2 Dexamethasone + lomustine chemotherapy: 1 Dexamethasone + whole-brain radiotherapy: 32 Dexamethasone + resection + whole-brain radiotherapy and ⁄ or stereotactic radiotherapy: 6	Not specified Hazard ratio of rectal cancer: 1.12 (95% CI: 0.8–2.87)	Median OS: 3.2. Median OS (potentially curative resection) = 13.2 (4.9–32.1) Dexamethasone only: 1.7 (0.4–5.4). Dexamethasone + shunt surgery to relieve hydrocephalus: < 1. Dexamethasone + whole-brain radiotherapy: 3.4 (1.0–11.2)
Chen 2022	2010–2018	195	91 M / 104 F	NA	SEER database	Patients with BM from CRC	Surgery, radiosurgery, whole-brain radiotherapy, and systemic therapy	Left colon: 46 (23.59%) Right colon: 83 (42.56%) Rectum: 66 (33.85%)	Median OS: 4.0 months
Chen 2023	2004–2020	65	32 M / 33 F	NA	Single Center: Taipei Veterans General Hospital	Patients with BM from CRC	Anti-VEGF based therapy: 25 (38.46%) None: 40 (61.54%)	Left colon: 55 (84.6%) Right colon: 10 (15.4%)	Median OS: 9.4 months
Michl 2015	1998–2011	228	134 M / 94 F	63 (27–94)	Munich Cancer Registry	Patients with BM from CRC	Not specified	Right colon: 49 (21%) Left colon + rectum: 155 (68%) Double/multifocal primary: 5 (2%) Unknown: 19 (8%)	Median OS: 2.0 months
Wang 2021	2009–2019	65	41 M / 24 F	63 (37–72)	Single Center: Second Affiliated Hospital of Zhejiang University	Patients diagnosed with CRC pathologically and BM were confirmed by radiologic imaging	Surgery: 28 (43.1%) Conservative: 37 (56.9)	Colon: 24 (36.9%). Rectal: 41 (63.1%)	Median OS: 11 months
Yang 2018	2010–2013	401	206 M / 195 F	NA	SEER database	Patients being treated with CRC presenting with BM	Not specified	Left colon: 92 right colon: 167 rectosigmoid: 139 appendix: 3	Median OS: 7 months (with metastasis to other organs), 15.59 months (without metastasis to other organs)

*CRC* colorectal cancer, *BM* brain metastasis, *OS* overall survival, *RT* radiotherapy, *CI* confidence interval, *SEER* *database* Surveillance, Epidemiology, and End Results database, *VEGF* vascular endothelial growth factor, *WBRT* whole-brain radiotherapy

### Comparative survival outcome

**Fig. 2:**
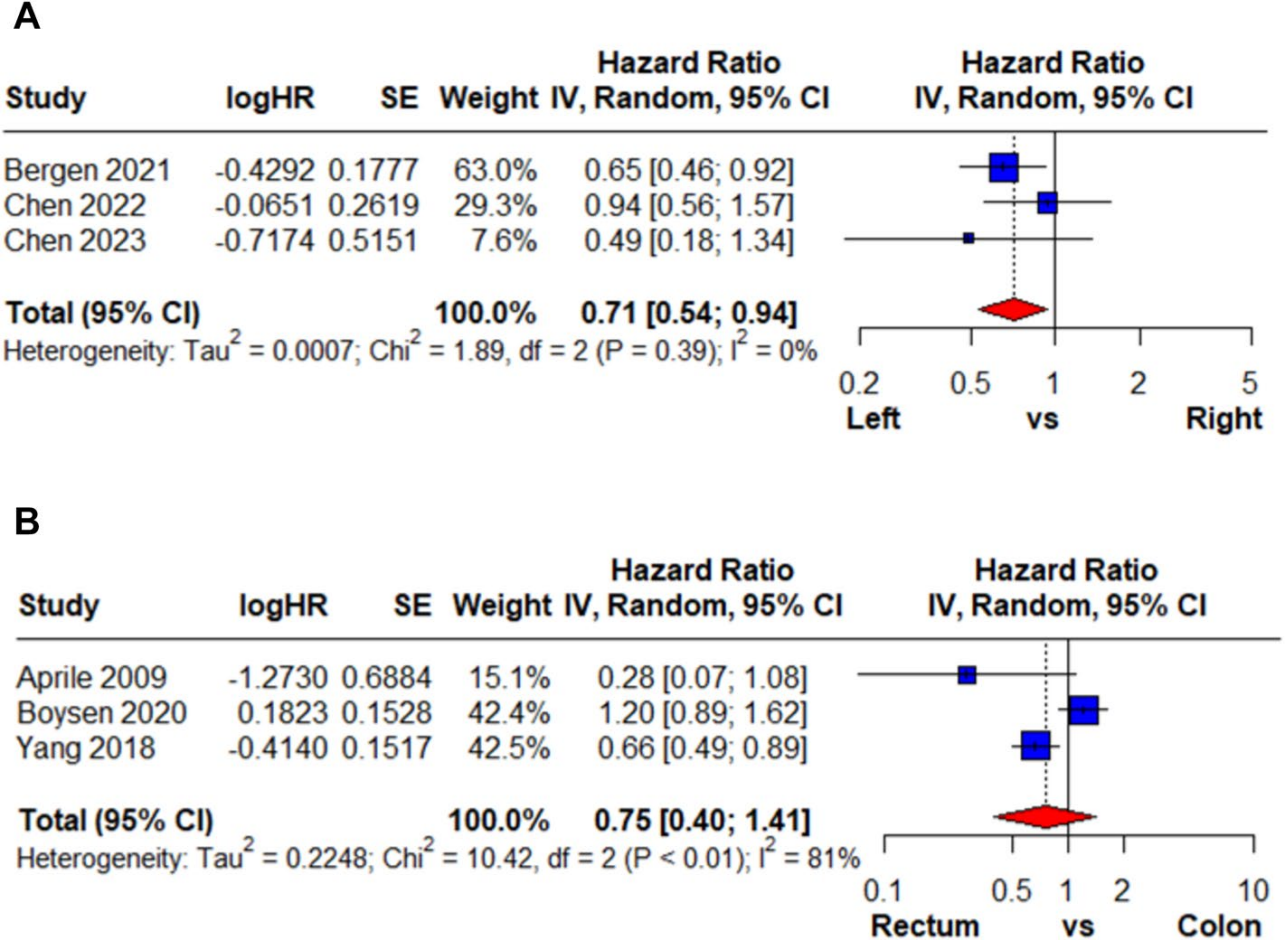
Multivariate meta-analysis results on the hazard ratio of CRCBM based on the primary cancer’s location

**Fig. 3:**
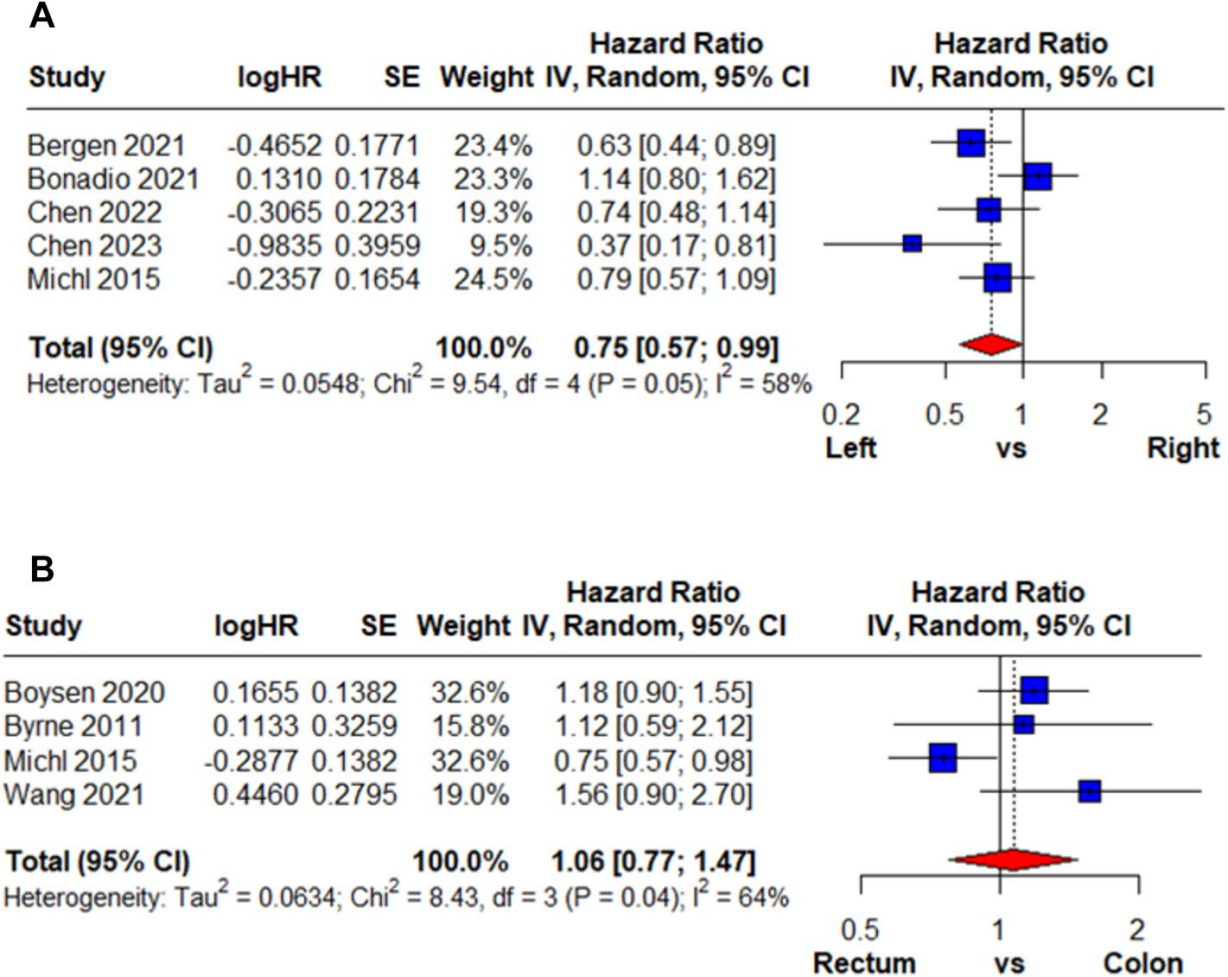
Univariate meta-analysis results on the hazard ratio of CRCBM based on the primary cancer’s location

HR was calculated in multivariate and univariate analyses for right-sided colon cancer versus left-sided colon cancer and colon cancer versus rectal cancer. For right-sided versus left-sided colon cancer, three studies using multivariate analysis were included (Fig. 2A). All individual study estimates favored left-sided tumors, and the pooled HR demonstrated a significant survival advantage (HR: 0.71, 95% CI: 0.54–0.94), with no heterogeneity (*I*^2^ = 0%). Pooled results from five studies using univariate analysis (Fig. 3A) yielded a similar pooled estimate (HR: 0.75, 95% CI: 0.57–0.99), with moderate heterogeneity (*I*^2^ = 58%). Overall, both models consistently indicated improved survival for left-sided colon cancer.

For colon versus rectal cancer, three studies using multivariate analysis were included (Fig. 2B), and pooled results showed no significant difference (HR: 0.75, 95% CI: 0.40–1.41) with substantial heterogeneity (I^2^ = 81%). Pooled results from four studies using univariate analysis (Fig. 3B) also demonstrated no survival difference (HR: 1.06, 95% CI: 0.77–1.47; *I*^2^ = 64%). The findings from both analytic approaches were inconsistent across studies. The overall results were not significant, indicating that rectal cancer did not provide a survival advantage compared with colon cancer.

### Quality assessment of the included studies

After performing a quality assessment of the included studies using the ROBINS-I tool, no critical risk of bias was identified (Supplementary Figure S4). Six studies were assessed as having a low risk of bias, and 3 studies were rated as having a moderate risk of bias. The remaining 1 study, Byrne 2011, was found to have a serious risk of bias, but it was not included in the multivariate analysis. [22]

## Discussion

Numerous studies have shown that primary tumor laterality in CRC significantly influences survival, with left-sided tumors generally demonstrating better outcomes than right-sided ones [4, 19, 21]. This prognostic distinction persists even in metastatic disease [6, 7]. For instance, Bingham et al. reported that right-sided CRC with liver metastases is associated with short overall, disease-free, and progression-free survival, even after hepatic management [7]. Similarly, Sasaki et al. found that left-sided CRC exhibits shorter disease-free intervals after hepatic resection, but recurrent disease originating from right-sided primary cancer is more advanced than that from left-sided primary cancer, resulting in reduced OS [25]. In our meta-analysis, despite performing subgroup analyses comparing right-sided versus left-sided and colon versus rectal primary tumors, substantial heterogeneity (*I*^2^ = 81%) persisted. This residual heterogeneity likely reflects unmeasured variations in treatment strategies, including surgery, radiotherapy, and systemic therapy, as well as differences in patient selection across the included studies.

Historically, CRC has been found to originate predominantly from the left colon, and the latest figures by the American Cancer Society confirm a higher overall proportion of left-sided CRC (54%) compared with right-sided CRC (39%) [26]. In general, right-sided CRCs are associated with a poor prognosis, primarily due to underlying genetic and histopathological characteristics [27, 28]. However, the prognostic significance of primary CRC location remained valid despite adjustment for underlying factors as reported by Loupakis et al. [29] Right-sided CRCs are predominantly sessile serrated adenomas and exhibit a flat morphology, which, unlike polypoid left-sided CRCs, can be overlooked during colonoscopy in earlier stages and often present at an advanced stage [27, 30]. In addition, the morphological dominance of mucinous adenocarcinomas results in a worse prognosis for right-sided CRCs as they advance more rapidly than adenomatous polyps [31]. One possible explanation includes the overexpression of mucin-2 (MUC-2) protein, which generally coats the intestinal epithelium and may inhibit antitumor effector immune responses, promoting tumor growth and progression. [31]

In terms of genomic makeup, right-sided CRCs have more microsatellite instability-high (MSI-high) tumors, whereas left-sided CRCs have chromosomal instability-high (CIN-high) tumors [27]. Tumors with MSI have a dysfunctional DNA mismatch repair (MMR) system due to germline mutations or inactivation of the MMR protein tumor suppressor genes, primarily MLH1, MSH2, and MSH6, or by epigenetic silencing of the MLH1 gene promotor by CpG island hypermethylation secondary to BRAF mutation [32, 33]. In addition, other tumor suppressor gene mutations in MSI-high tumors include MSH3, POLE, APC, ACVR2A, and TGFβR2 [27]. The oncogene BRAF mutation commonly seen with right-sided CRC and MSI tumors is a prognostic indicator for OS and confers a poor prognosis [33, 34]. These mutations cause MSI-high tumors to possess an increased number of neoantigens, facilitating greater T-cell infiltration [27], which may confer advantages in OS and relapse-free survival for right-sided CRC; however, the general prognosis for right-sided CRC remains poor due to other factors described herein [28, 35]. By contrast, left-sided CRC usually develops via a traditional CIN-high pathway, presenting with aneuploidy and loss of heterozygosity in chromosomes [27]. The adenoma–carcinoma sequence of events in left-sided CRC carcinogenesis begins with the loss of the APC tumor suppressor gene followed by KRAS oncogene activation, p53 tumor suppressor gene inactivation, and other mutations in tumor suppressor (FBXW7, SMAD2, SMAD4, TCF7L2, FAM123B (WTX), ATM, and ARID1A) genes and oncogenes (PIK3CA, NRAS, CTNNB1, and SOX9) [27]. These genetic differences explain the varying tumor biology of right- and left-sided CRC, and they may also mechanistically influence survival following BM. MSI-high and BRAF-mutated right-sided tumors possess higher mutational burdens and generate abundant neoantigens compared with their counterparts, potentially enhancing immune cell infiltration within primary and metastatic sites. However, despite this immunogenic profile, such tumors often exhibit aggressive clinical behavior, early systemic dissemination, and limited responsiveness to conventional chemotherapy, resulting in inferior overall outcomes after BM. Conversely, left-sided CRCs with CIN-high signatures follow a gradual adenoma–carcinoma sequence with relatively stable genomic evolution, which may result in delayed metastatic progression and improved survival upon the onset of BM.

As a result of differing characteristics, CRC treatment responses vary by location. MSI-high tumors exhibit low response rates to 5-fluorouracil (5-FU) chemotherapy, especially in right-sided CRCs, attributed to loss of MMR genes MLH1 and MSH2 [36]. Right-sided CRCs also show high chemoresistance to FOLFOX due to increased ERCC-1 expression and poor response to 5-FU due to elevated DNA topoisomerase 1 [29, 31]. Immunotherapies targeting checkpoint inhibitors like PD-1, PD-L1, and CTLA-4 can effectively target chemoresistant right-sided CRCs by utilizing their lymphocyte-rich microenvironments [27, 37]. By contrast, left-sided CRCs, with high expression levels of epiregulin and amphiregulin, exhibit relative chemo-sensitivity, distinguishing them from BRAF-mutant right-sided CRCs. [29, 38]

One meta-analysis on the OS of CRC with BM, regardless of the primary tumor site, indicated that the incidence of BM in CRC is rare, but the survival outcomes are poor [39]. Most patients (96.8%) were asymptomatic at the time of BM diagnosis, which, together with a lack of formal screening guidelines, resulted in a delayed diagnosis [39]. In addition, evaluating outcomes in CRC with BM presents challenges due to the blood–brain barrier, affecting the efficacy of treatments like monoclonal antibodies [40]. BM complicates CNS involvement, causing symptoms such as tumor mass effects, hydrocephalus, and seizures. These neurological complications independently contribute to survival variations, potentially overshadowing the primary tumor site’s direct influence [39, 40]. Nonetheless, the survival outcomes remained poor with right-sided CRC with BM, suggesting that the intrinsic characteristics of primary CRC dictate prognosis rather than metastases.

This study demonstrated that left-sided CRC exhibited a better OS compared with right-sided CRC in multivariate and univariate analyses. However, when comparing colon cancer with rectal cancer, no significant differences were observed among the primary locations. This lack of distinction may be attributed to left-sided CRC being anatomically and histologically more similar to rectal cancer than right-sided CRC [1]. Several analyses from the SEER database presented comparisons between right colon cancer and rectal cancer, consistent with our analysis of right-sided CRC versus left-sided CRC. For example, Yang showed superior OS of rectal cancer compared with right colon cancer (HR: 0.654, 95% CI: 0.631–0.679). [16]

This study had several limitations. First, several studies were excluded from our study because they did not provide HRs for each location of primary CRC. Second, the secondary outcome of the study, comparing colon cancer with rectal cancer, exhibited high heterogeneity in multivariate and univariate analyses. This variability may be attributed to the lack of subdivision into right-sided, left-sided, or rectal CRCs, as well as differences in treatment modalities. In the studies by Aprile and Del Carpio Huerta, patients underwent surgery with or without postoperative radiotherapy [18, 25]. In the study by Chen, patients received anti-VEGF therapy [14]; for most of the studies, they used multiple methods including conservative treatment or chemotherapy only. Lastly, all included studies were retrospective cohort studies and lacked detailed molecular data by tumor location, introducing potential confounding and bias. Moreover, several studies were derived from the SEER database, suggesting the potential for data overlap and selection bias. Nevertheless, this work is the first systematic review and meta-analysis to evaluate the influence of primary tumor location on survival in patients with CRCBM.

## Conclusion

Consistent with previous studies, when metastasized to the brain, left-sided colon cancer showed higher OS compared with right-sided colon cancer. Further studies comparing the genotypes and mutations of CRCBM based on their primary location are needed.

## Supplementary Information

Additional file 1: Table S1. PRISMA 2020 checklist. Table S2. Search strategy for Embase, Medline, and Cochrane. Supplementary S3. Data synthesis methodology. Figure S4. ROBINS-I tool for quality assessment of included studies.

## Data Availability

As this study is a meta-analysis, the data is accessible through a PubMed search. Detailed data will be shared upon reasonable request to the corresponding author.
